# Region-specific astrogliosis: differential vessel formation contributes to different patterns of astrogliosis in the cortex and striatum

**DOI:** 10.1186/s13041-020-00642-0

**Published:** 2020-07-22

**Authors:** Haijie Yang, Jiawei An, Insup Choi, Kihwang Lee, Sang-Myun Park, Ilo Jou, Eun-Hye Joe

**Affiliations:** 1 Department of Pharmacology/Neuroscience Graduate Program, National Research Lab of Brain Inflammation, Ajou University School of Medicine, Worldcup-ro 164, Youngtong-gu, Suwon, Kyunggi-do 16499 South Korea; 2 Department of Brain Science, Worldcup-ro 164, Suwon, Kyunggi-do 16499 South Korea; 3 Department of Biomedical Sciences, Neuroscience Graduate Program, Worldcup-ro 164, Suwon, Kyunggi-do 16499 South Korea; 4 Department of Ophthalmology, Worldcup-ro 164, Suwon, Kyunggi-do 16499 South Korea; 5 Chronic Inflammatory Disease Research Center, Ajou University School of Medicine, Worldcup-ro 164, Suwon, Kyunggi-do 16499 South Korea

**Keywords:** Astrocyte, Glial scar, Brain injury, Cortex, Striatum

## Abstract

Brain injury causes astrocytes to become reactive (astrogliosis). In this study, we compared astrogliosis in acutely injured cortex and striatum of adult FVB/N mice induced by stereotaxic injection of ATP, a component of danger-associated molecular patterns (DAMPs). Interestingly, MR analysis showed that same amount of ATP induced smaller damage in the cortex than in the striatum. However, in histological analysis, thick and dense scar-like astrogliosis was found in the injured cortex near meninges within 2 wk., but not in other regions, including the striatum and even the cortex near the corpus callosum for up to 30 d. There was little regional difference in the number of Ki67(+)-proliferating astrocytes or mRNA expression of inflammatory cytokines. The most prominent difference between regions with and without scar-like astrogliosis was blood vessel formation. Blood vessels highly expressing collagen 1A1 formed densely near meninges, and astrocytes converged on them. In other regions, however, both blood vessels and astrocytes were relatively evenly distributed. Consistent with this, inhibition of blood vessel formation with the vascular endothelial growth factor (VEGF)-blocking antibody, Avastin, attenuated scar-like astrogliosis near meninges. These results indicate that region-specific astrogliosis occurs following brain injury, and that blood vessel formation plays a critical role in scar formation.

## Introduction

Astrocytes in the injured brain undergo astrogliosis, characterized by a hypertrophic morphology and increased expression of glial fibrillary acidic protein (GFAP) [1, 2]. Astrogliosis is not an all-or-none phenomenon. Instead, it manifests as mild to severe changes that have been suggested to depend on the extent of the injury. In the severely injured brain, astrocytes form irreversible scars [3], which are considered to act as a barrier that inhibits axon regeneration in the injured spinal cord and brain. However, it has recently been suggested that scar formation has beneficial effects on the repair of the injured brain [4].

In addition to astrocytes, several types of cells and/or molecules contribute to astrogliosis and/or scar formation. Microglia trigger astrocyte activation through production of cytokines [3, 5]. Glia and pericytes expressing neuron-glial antigen 2 (NG2), also known as chondroitin sulfate proteoglycan 4 (CSPG4), also contribute to scar formation [6–8]. CSPGs are well-known components of scar [9, 10]. It has recently been reported that type I collagen expressed in pericytes increases during scar formation and that interaction of astrocytes with type I collagen induces astrocytic scars [11]. In addition to these positive regulators of scar formation, monocytes that infiltrate from blood into the injured brain negatively regulate scar formation by secreting matrix metalloproteinase 13 (MMP-13), which degrades CSPGs [12, 13]. Astrocytes and microglia in different regions of the intact and injured brain exhibit differences in phenotypes, densities, and/or functions [14–19]. In addition, the distribution of blood vessels and infiltration of blood cells into the injured brain differ in different brain regions [17, 18, 20] . These observations raise the question of whether the pattern of astrogliosis in response to injury may be different in different brain regions.

In this study, we demonstrate region-specific differences in astrogliosis, showing that scar-like dense astrogliosis occurred in the cortex near the meninges, but not in the cortex near the corpus callosum and the striatum. In addition, blood vessel formation was heaviest near meninges, and blocking vessel formation mitigated scar-like astrogliosis, suggesting that blood vessel formation contributes to the formation of scar-like dense astrogliosis.

## Materials and methods

### Animals

FVB/N mice (male, 8–10 wk. old, 25–30 g) were housed under a 12-h light/dark cycle with free access to food and water. All experiments were performed in accordance with approved animal protocols and guidelines established by the Ajou University School of Medicine Ethics Review Committee (2014–0029; AMC119).

### Stereotaxic surgery and drug administration

Mice were anesthetized by intraperitoneal injection of 2.5% Avertin (2,2,2-tribromoethanol and *tert*-amyl alcohol) at a dose of 0.015 ml/g body weight, and placed into a stereotaxic apparatus (David Kopf Instruments, Tujunga, CA, USA). ATP, previously established as a suitable insult for mimicking pathological conditions [21–24], was used to induce brain damage. Specifically, 0.8 μl of ATP (500 mM) was administered into the cortex (AP, + 1.0; ML, + 1.6; DV, − 1.1) and striatum (AP, + 1.0; ML, − 1.9; DV, − 3.2), according to the atlas of Paxinos and Watson, using a Hamilton syringe equipped with a 33-gauge needle attached to a syringe pump (KD Scientific, New Hope, PA, USA). The injection rate was 0.2 μl/min, and the needle was left in place for an additional 8 min prior to removal to prevent leakage through the needle track.

To inhibit vessel formation, the anti-VEGF monoclonal antibody, Avastin (bevacizumab; Genentech BioOncology, South San Francisco, CA, USA), was infused into the lateral ventricle (10 mg/kg, 0.25 μl/h) for 14 d using ALZET mini osmotic pumps (DURECT Corporation, Cupertino, CA, USA) as described previously [25, 26]. Briefly, a metal cannula was inserted into the ventricle (AP: -0.5, ML: + 1.2, DV: − 2.5) in the side contralateral to ATP injection, and the minipump was placed into a subcutaneous pocket. Infusion started immediately after finishing ATP injection into the cortex.

### Magnetic resonance imaging (MRI)

Changes in brain damage was chased for 15 d using a 9.4 T MR scanner (BioSpec 94/30 US/R; Bruker, Billerica, MA, USA) at Sungkyunkwan University (Suwon, Korea). Briefly, mice were anesthetized with 1.5% isoflurane. Respiration and body temperature were continuously assessed using an MR-compatible small animal monitoring and gating system (Model 1025; SA Instruments, Inc., Stony Brook, NY, USA). T2-weighted 2D Turbo rapid imaging with a refocused echo (RARE) sequence was performed using the following parameters: repetition time (TR) = 9000 ms; echo time (TE) = 33 ms; resolution, 78 μm × 78 μm × 250 μm; number of slices, 25; thickness, 250 μm; RARE factor, 8; average, 2; scan time, 10 min. MR images were analyzed using Mimics software (Materialise, Leuven, Belgium).

### Tissue preparation

Mice were anesthetized and transcardially perfused with phosphate-buffered saline (PBS) containing 0.5% sodium nitrate and heparin (10 U/ml), and then with 4% paraformaldehyde in 0.1 M phosphate buffer (PB; pH 7.2). Brains were stored at 4 °C in 4% paraformaldehyde in 0.1 M PB for 1 d, and then kept in 30% sucrose in PBS until they sank. For immunostaining, six separate series of 35-μm-thick coronal sections were obtained using a cryostat (Leica, Wetzlar, Germany) and stored in an antifreeze stock solution (PB containing 30% glycerol and 30% ethylene glycol, pH 7.2) at 4 °C before use.

For protein and RNA preparation, brains were obtained after transcardial perfusion with PBS. A brain slice (2 mm thickness) including the needle injection site was prepared using an Alto mouse brain slicer matrix (Roboz Surgical Instruments, Gaithersburg, MD, USA). Tissue blocks (2 × 2 × 2 mm^3^) were collected and stored at − 70 °C until use.

### Immunostaining

For 3,3′-diaminobenzidine (DAB) staining, every sixth serial section in each set (~ 8 sections) was collected, rinsed three times with PBS, treated with 3% H_2_O_2_ for 5 min, and rinsed again with PBS. After incubation with 1% bovine serum albumin (BSA) in PBS, the sections were incubated overnight at 4 °C with primary antibodies (Table 1). The sections were rinsed three times with PBS and incubated with biotinylated secondary antibodies (Vector Laboratories, Burlingame, CA, USA). Immunoreactive proteins were visualized using an avidin-biotin-peroxidase-DAB solution (0.05% DAB and 0.003% H_2_O_2_ in 0.1 M PB) according to the manufacturer’s instructions. The sections were mounted on slides and examined under a bright-field microscope (Olympus, Tokyo, Japan). Images were captured using PictureFrame Application 2.3 software. Photographs of the most damaged sections are presented in the results.

**Table 1:** Antibodies used in this study

Antibodies	Dilution factors for	
	Western blot	Immunostaining
Rabbit anti-ALDH1L1 (Abcam, ab87117)	1:1000	1:100
Rabbit anti-GFAP (Neuromics, RA22101)		1:1000
Rabbit anti-Col1a1 (Novus, NB600–408)		1:200
Rabbit anti-Ki67 (Chemicon, AB9260)		1:100
Mouse anti-GFAP (Sigma-Aldrich, G3893)	1:5000	1:1000
Goat anti-GAPDH (Santa cruz, sc-48167)	1:5000	
Rat anti-CD45 (Mybiosource, MBS520149)		1:1000
Rabbit anti-Iba-1 (Wako, 019–19741)		1:1000
Rat anti-CD31 (BD Pharmingen, 550274)		1:50
Chicken anti-MAP2 (Abcam, ab5392)		1:500
Rabbit anti-TH (Pel Freez, P40101–0)		1:1000
DAPI (Sigma-Aldrich, D9542)		1:2000
Goat anti-rabbit IgG HRP (Koma biotech, K0211708)	1:10000	
Goat anti-mouse IgG HRP (Koma biotech, K0211589)	1:10000	
Rabbit anti-Goat IgG HRP (Koma biotech, K0211303)	1:10000	
Goat Anti-rabbit IgG (H + L) (Vector Laboratories, BA-1000)		1:200
Goat Anti-rat IgG (H + L) (Vector Laboratories, BA-9401)		1:200
Goat anti-mouse IgG, Alexa Fluor 488 (Thermo Fisher scientific, A-11029)		1:500
Goat anti-chicken IgY, Alexa Fluor 488 (Thermo Fisher scientific, A-11039)		1:500
Goat anti-rabbit IgG, Alexa Fluor 594 (Thermo Fisher scientific, A-11037)		1:500
Goat anti-rat IgG, Alexa Fluor 633 (Thermo Fisher scientific, A-21094)		1:500
Goat anti-mouse IgG, Alexa Fluor 568 (Thermo Fisher scientific, A-11004)		1:500

For immunofluorescence staining, sections were washed with PBS containing 0.1% Triton X-100 (PBST), treated with 1% BSA, and incubated with combinations of primary antibodies (Table 1). Immunoreactive proteins were visualized using Alexa Fluor 488-, Alexa Fluor 568-, Alexa Fluor 594-, or Alexa Fluor 633-conjugated secondary antibodies (1:500 dilution; Invitrogen, Carlsbad, CA, USA). Nuclei were visualized using 4′,6-diamidino-2-phenylindole (DAPI, 0.5 μg/ml; Sigma). Sections were embedded in Fluoroshield Mounting Medium (Abcam, Cambridge, Great Britain). Images were captured using an LSM 800 confocal microscope (Carl Zeiss, Oberkochen, Germany) and analyzed with ZEN software (Carl Zeiss, Oberkochen, Germany). Additionally, intensity profiles of the immunofluorescence staining in the subareas of the images were measured using Zen software.

### Image analysis

Astrocytes were assessed by immunostaining for GFAP, a specific marker of astrocytes. The lengths of astrocyte processes were measured using MetaMorph Image Analysis software (Molecular Devices, San Jose, CA, USA). DAB-stained GFAP images were acquired on a light microscope (Olympus BX51, Tokyo, Japan) at 20x magnification and saved as 24-bit TIFF images. Process lengths of the first row of astrocytes that surround the damage area were analyzed. For each time point, approximately 150 astrocytes were analyzed from 4 to 5 animals, and 4–5 ROIs in each animal.

For quantification of astrocyte proliferation, sections were stained with antibodies for Ki67 and GFAP, and images were obtained with a confocal microscope (Zeiss 800). Proliferating cells were counted using Imaris 9.3.1 software (Bitplane, Belfast, UK).

### Total protein extraction and Western blotting

Total protein was extracted on ice using RIPA buffer (10 mM PB pH 7.2, 150 mM NaCl, 1% NP-40, 0.5% sodium deoxycholate) containing a protease/phosphatase inhibitor cocktail (GenDEPOT, Barker, TX, USA). Proteins were denatured by incubating for 10 min at 95 °C in sample buffer (6.25 mM Tris pH 6.8, 12.5% glycerol, 2.5% SDS, 0.025% bromophenol blue, and 5% β-mercaptoethanol), separated by sodium dodecyl sulfate-polyacrylamide gel electrophoresis (SDS-PAGE), and transferred to a nitrocellulose membrane (GE Healthcare, Pittsburgh, PA, USA). After blocking with 5% skim milk (Seoul Dairy Coop, Seoul, Korea), the nitrocellulose membrane was sequentially incubated with primary antibodies (Table 1), peroxidase-conjugated secondary antibodies (Koma Biotech, Seoul, Korea), and enhanced chemiluminescence reagents (Daeil Lab Services, Seoul, Korea). Glyceraldehyde 3-phosphate dehydrogenase (GAPDH) was used as a loading control.

### Quantitative real-time polymerase chain reaction (QPCR)

Total RNA was isolated from injured mouse brains using easy-BLUE reagent (iNtRON Biotechnology, Seongnam-si, Korea). cDNA was synthesized from 1 μg of total RNA using a cDNA synthesis kit (iNtRON Biotechnology) following the manufacturer’s guidelines. Each RNA sample was run in triplicate, and each group consisted of three to five animals. cDNA and forward/reverse primers (1 μM) were mixed with 2X Kapa SYBR Fast Master Mix (Kapa Biosystems, Boston, MA, USA), and quantitative real-time reverse transcription-polymerase chain reaction (RT-qPCR) was carried out using a Corbett Rotor Gene-6000 real-time amplification instrument (Corbett Research, Sydney, Australia). Threshold cycle values were calculated for each gene and normalized to that of GAPDH, used as an internal reference. Details of primer sequences and product sizes are presented in Table 2.

**Table 2:** Primer sequences for real-time qPCR analysis

mRNA	Oligonucleotide Sequence (5′-3′)	Amplicon^a^ (bp)	Gene Bank No.^b^
IL-1β	F: TCC AGG ATG AGG ACA TGA GCA C R: GAA CGT CAC ACA CCA GCA GGT TA	105	NM_008361.3
IL-6	F: CCA CTT CAC AAG TCG GAG GCT TA R: CCA GTT TGG TAG CAT CCA TCA TTT C	169	NM_031168.1
TNF-α	F: CTT CTG TCT ACT GAA CTT CGG R: CAG GCT TGT CAC TCG AAT TTT	134	NM_013693.3
CD45	F: TGG AAT GAC CTC AAG GTG TCC TC R: GCT GTA CAC ACC CAC AGC ACT CTT	108	NM_001111316.2
MMP-13	F: TTC TTG TTG AGC TGG ACT CCC TGT R: TGC TCT GCA AAC ACA AGG TCT TCC	98	NM_008607.2
GADPH	F: GCC TTC CGT GTT CCT ACC R: CCT CAG TGT AGC CCA AGA TG	142	NM_001289726.1

^a^Predicted number size of PCR product; ^b^NCBI reference sequence

### Statistical analysis

Statistical analyses were performed using GraphPad Prism 7 software (GraphPad; San Diego, CA, USA). Comparison of damaged volumes in MRI was analyzed using repeated measures ANOVAs with Geisser-Greenhouse correction and Sidák post hoc analyses with corrections for multiple comparisons. Similar ANOVAs and post hoc analyses were used to examine differences in mRNA level and process length. Comparisons of protein levels in several brain regions were analyzed using ordinary one-way ANOVA. Comparisons of two groups between the cortex and the striatum were assessed using unpaired Student’s t-tests. All values are means ± SEMs of at least three independent experiments.

## Results

### Region-specific astrocyte responses to injury

**Fig. 1:**
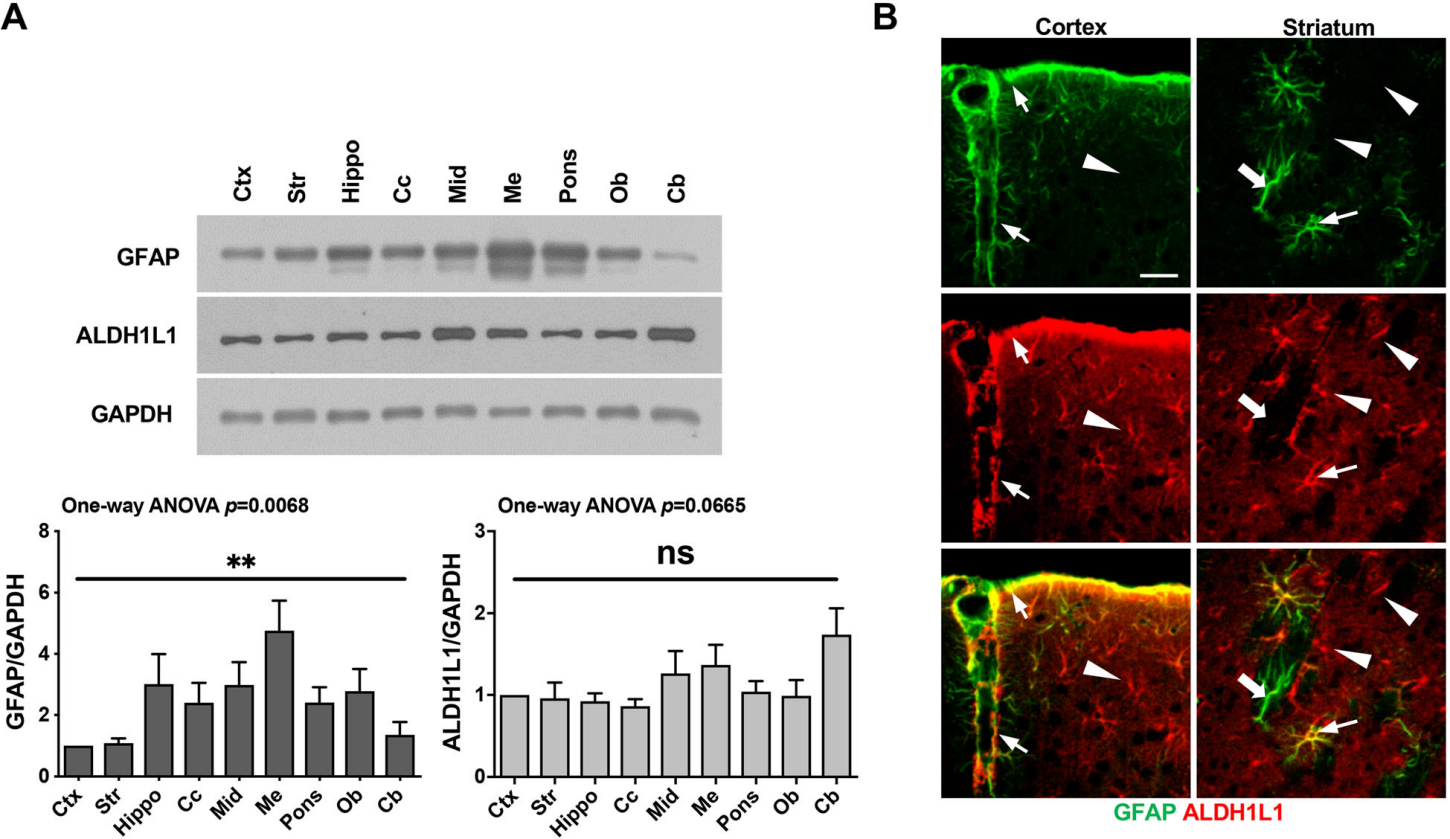
Differential expression of the astrocyte markers, GFAP and ALDH1L1, in different brain regions. **a** Tissue lysates were prepared from several brain regions. GFAP and ALDH1L1 protein levels in each brain region were analyzed by Western blotting. GAPDH was used as a loading control. Ctx, cerebral cortex; Str, striatum; Hippo, hippocampus; Cc, corpus callosum; Mid, midbrain; Me, medulla; Ob, olfactory bulb; and Cb, cerebellum. Band intensities were analyzed using Image J. Values are means ± SEMs of three samples (ns, not significant; **, *p* < 0.001; one-way ANOVA, GFAP: F (8, 36)=3.261, *p* = 0.0068; ALDH1L1: F (8, 36)=2.060, *p* = 0.0665). **b** Sections obtained from the cortex and striatum were stained with antibodies against GFAP and ALDH1L1, and immunoreactive proteins were visualized using Alexa 488- and 594-conjugated secondary antibodies. Three types of astrocytes were detectable in the cortex and the striatum: GFAP and ALDH1L1 double-positive (thin arrows), GFAP-positive/ALDH1L1-negative (thick arrows), and ALDH1L1-positive/GFAP-negative (arrowheads). Scale bar, 10 μm. Data are representative of at least 3 animals

To analyze the region-specific responses of astrocytes to injury, we selected the cortex and striatum, two brain regions where GFAP levels were similar (Fig. 1a). Expression levels of the astrocyte marker, GFAP, were relatively low in the cortex and striatum compared with other brain regions, including the olfactory bulb and hippocampus, among others, whereas that of another astrocyte marker, ALDH1L1, were largely similar in all brain regions examined (Fig. 1a). Immunostaining, however, showed diverse astrocytes in the cortex and the striatum: GFAP and ALDH1L1 double-positive (thin arrows), GFAP-positive/ALDH1L1-negative (thick arrows), and ALDH1L1-positive/GFAP-negative (arrowheads) (Fig. 1b).

**Fig. 2:**
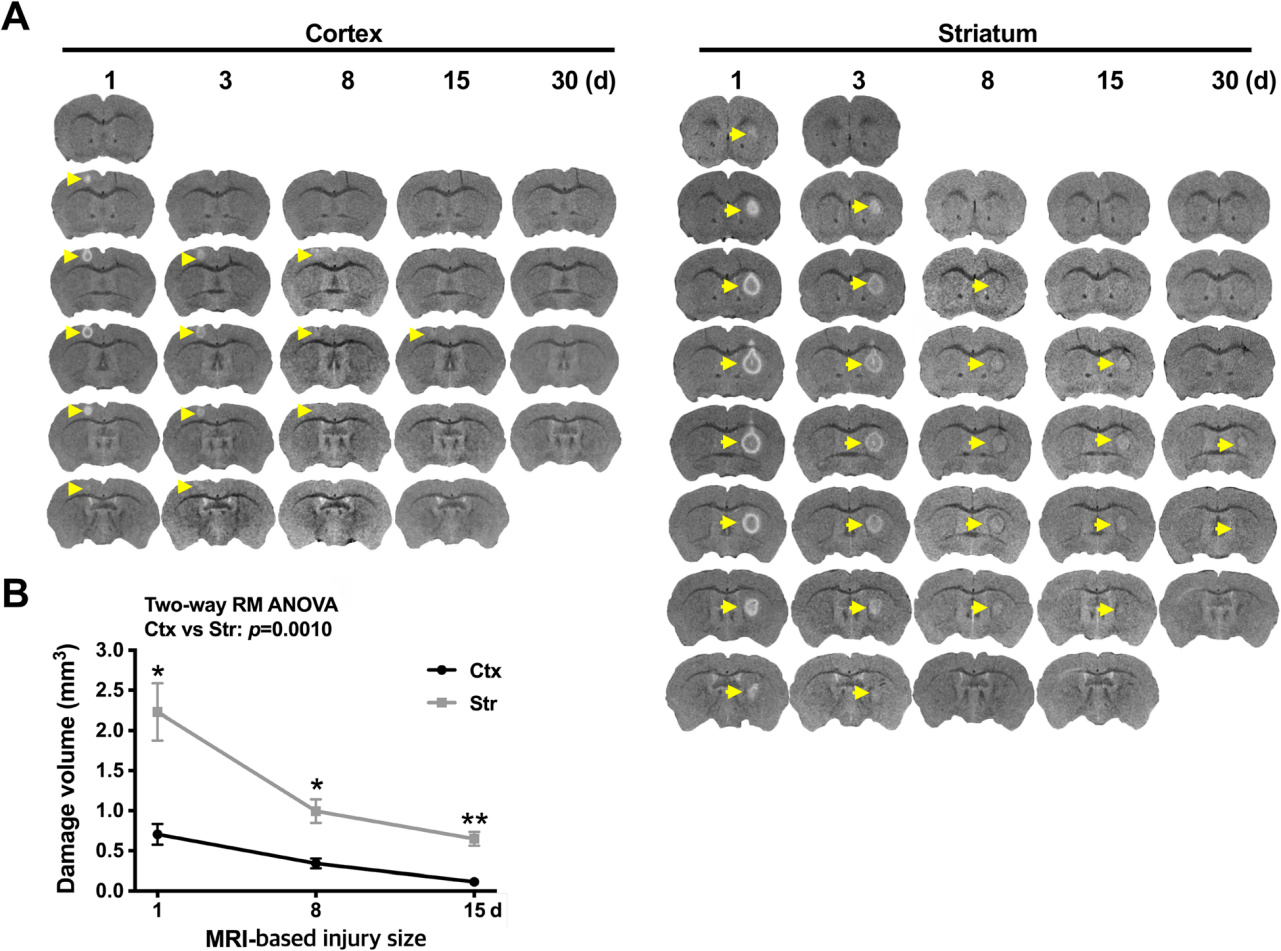
MR analysis of region-specific brain injury. **a** Brain damage was induced by stereotaxic injection of ATP (400 nmole) into the cortex or striatum. As described in Methods, changes in damage were chased using a 9.4 T MR apparatus for 15 d, at the indicated times after ATP injection. T2-MR images were obtained every 250 μm. **b** Damage volume was quantified using Mimics software. Values are means ± SEMs of 6 (cortex) or 7 (striatum) animals (**, *p* < 0.001 for cortex vs. striatum; two-way ANOVA, F (2, 22)=7.6, *p* = 0.0029, interaction; F(1.064,11.70 = 33.26, *p* < 0.0001, time; F (1, 11)=19.43, *p* = 0.0010, region; F (11, 22)=3.313, *p* = 0.0081, subjects)

Brain damage was produced by stereotaxic injection of ATP (400 nmole), a component of DAMPs that induces acute brain injury [21–24], and monitored using 9.4 T MRI, with damage sites appearing white in T2-MR images (arrows in Fig. 2a). Although the same amount of ATP was injected into the cortex and the striatum, T2-MR images obtained 1 d after ATP injection showed that the damage volume in the cortex (0.71 ± 0.13 mm^3^) was smaller than that in the striatum (2.23 ± 0.36 mm^3^). Damage volumes decreased in both regions (more rapidly in the striatum) for up to 30 d (Fig. 2a, b). At 30 d, damage sites were still detectable in the striatum, but not in the cortex (arrows in Fig. 2a).

**Fig. 3:**
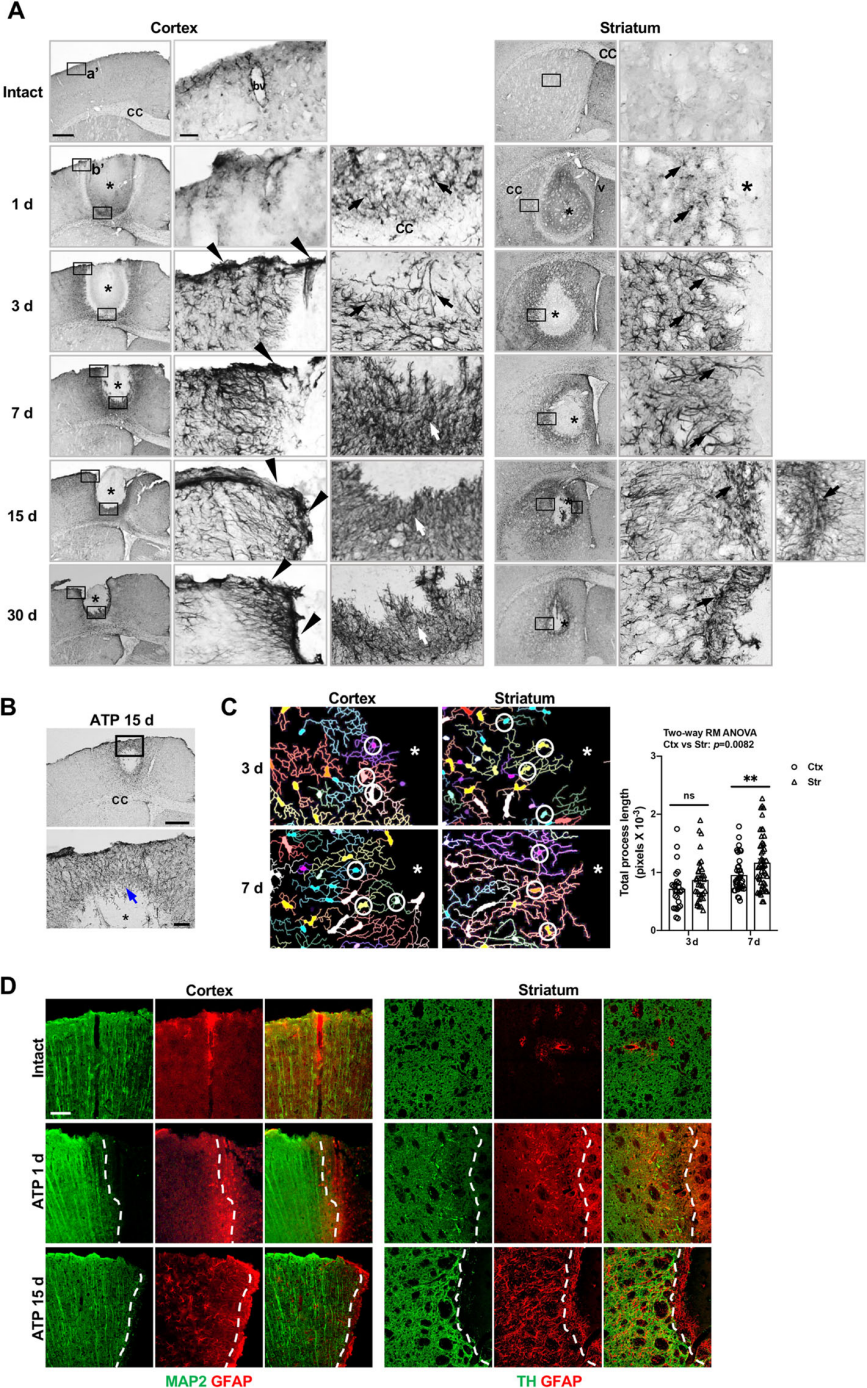
Different patterns of astrogliosis in the cortex and striatum. **a, b** Coronal sections were obtained from the intact or ATP (400 nmole)-injected cortex or striatum at the indicated times after injection, and immunostained for GFAP. Photographs of the most damaged sections in the injured brain are shown. *, injection sites; arrowheads and arrows, GFAP-positive cells in different brain regions; cc, corpus callosum; bv, blood vessel; v, lateral ventricle. **c** Process lengths of astrocytes adjacent to the damage core (circles) were analyzed 3 and 7 d post injury using MetaMorph as described in the Methods. For each time point, 4–5 animals were used. Values are means ± SEMs. n, approximately 150; *, *p* < 0.05; ns, not significant; two-way ANOVA, F(1,140) = 15.41, *p* = 0.0001, time; F(1,140) = 7.189, *p* = 0.0082, region. *, injection sites. **d** Sections obtained from the intact or ATP-injected cortex or striatum 1 and 15 d post injection, and immunostained for MAP2/GFAP or TH/GFAP. Dotted lines, borders of MAP2- and TH-positive neurites; *, injection sites

In this ATP-induced injury model, immunostaining using GFAP antibodies revealed different dynamic behaviors of astrocytes in the cortex and the striatum (Fig. 3). As expected, GFAP expression progressively increased in the penumbra region of the damage core at all injury sites (Fig. 3a). However, astrogliosis occurred very densely in the cortex near the meninges from 3 d to 30 d (arrowheads in Fig. 3a), but was relatively less dense in the cortex near the corpus callosum and the striatum (arrows in Fig. 3a). Interestingly, however, damage in the cortex in the absence of meninges damage produced a degree of astrogliosis similar to that in the injured cortex near the corpus callosum and striatum (arrows in Fig. 3b). The lengths of astrocyte processes progressively increased from 3 d to 7 d, and were longer in the striatum than in the cortex at 7 d, particularly that of astocytes adjacent to the damage core (Fig. 3a, c). Neurites also grew in the injured cortex and striatum together with astrocytes. Microtubule associated protein 2 (MAP2) immunoreactivity in the cortex, and tyrosine hydroxylase (TH) immunoreactivity in the striatum, were detectable near, but slightly behind astrocytes 1 d and 15 d post injury (dotted lines in Fig. 3d). Taken together, these findings clearly demonstrate that astrocytes differentially respond to injury in different brain regions.

### Patterns of astrocyte proliferation and inflammation in the injured cortex and striatum

**Fig. 4:**
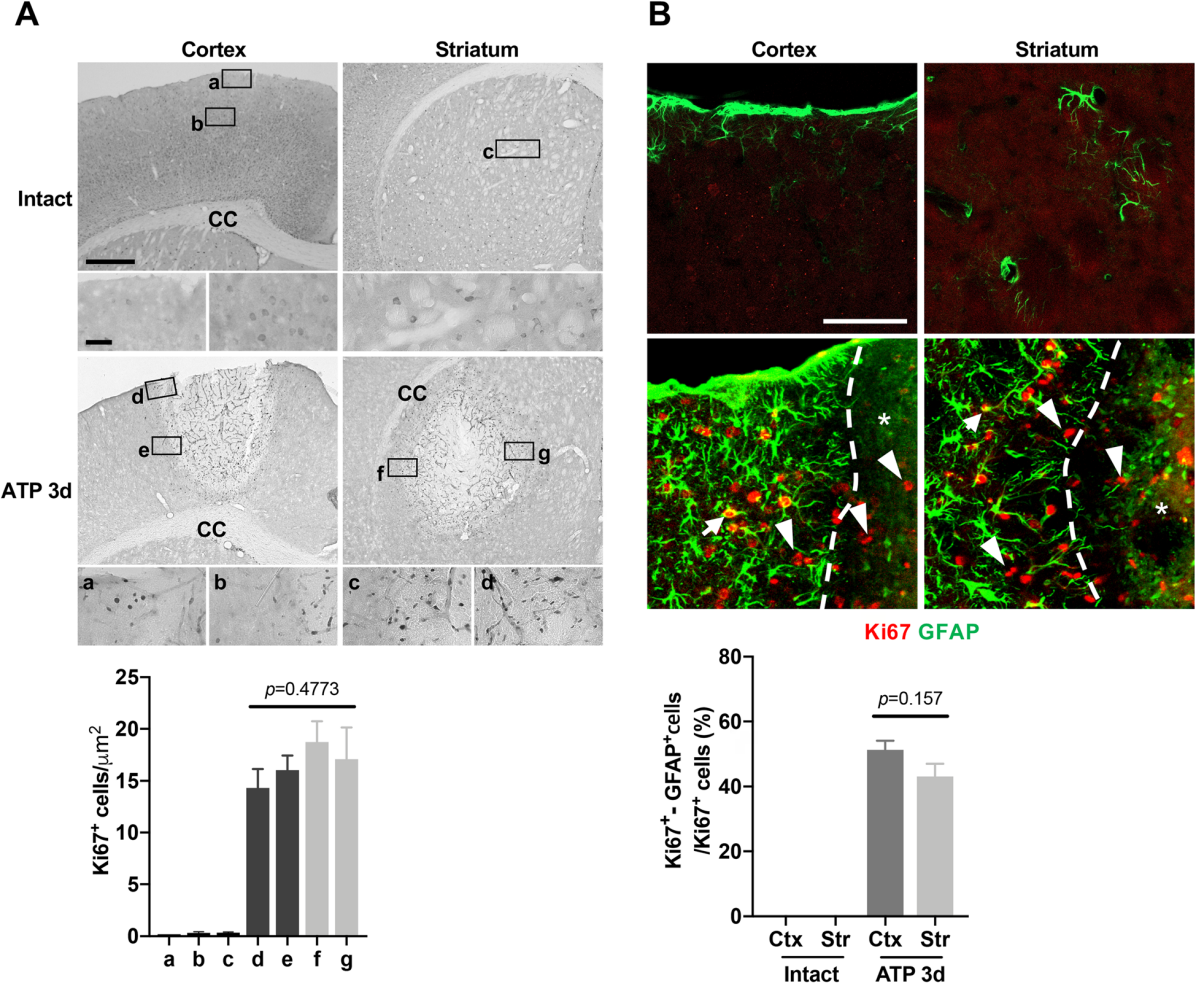
Cell proliferation is not different between the injured cortex and striatum. **a, b** Upper panel: Sections obtained from the intact and or 3 d post injury were stained with antibodies for Ki67 (**a**) and GFAP and Ki67 (**b**). Ki67/GFAP double-positive cells (arrows) and Ki67-positive/GFAP-negative cells (arrowheads) were found in the P1 region and damage core. Dotted lines in (**b**), edges of damage areas. Lower panel: For quantification of astrocyte proliferation, sections were stained with antibodies for Ki67 and GFAP. With Imaris software, the number of Ki67/GFAP double-positive cells and Ki67-positive/GFAP-negative cells were analyzed in 4 (cortex) or 6 mice (striatum), and 3 sections from each mouse. Values are means ± SEMs. ns, not significant; **, *p* < 0.001; one-way ANOVA, F (3, 25)=0.8549, *p* = 0.4773 (**a**); Student’s t-test, F (3, 5)=2.766, *p* = 0.1571 (**b**)

It has been reported that proliferation of astrocytes and/or NG-2–expressing cells contributes to astrogliosis [6, 27, 28]. Accordingly, we examined the proliferation of astrocytes in the cortex and striatum using antibodies for Ki67, a proliferation marker. Ki67-positive cells were not detectable in intact cortex and striatum, but detectable in the core and surrounding regions of the injury (Fig. 4Aa-c). Unexpectedly, however, the number of Ki67-positive cells was not different among four different regions of interest: cortex near the meninges (Fig. 4Ad), cortex near the corpus callosum (Fig. 4Ae), striatum near the corpus callosum (Fig. 4Af), and striatum near the ventricle (Fig. 4Ag). Double-immunostaining showed that Ki67 was detectable in GFAP-positive (arrows) and -negative cells in the cortex and striatum of injured but not in intact brains (arrowheads, Fig. 4b, upper panel). In addition, the number of Ki67/GFAP-double–positive cells was similar in both regions (Fig. 4b, lower panel).

**Fig. 5:**
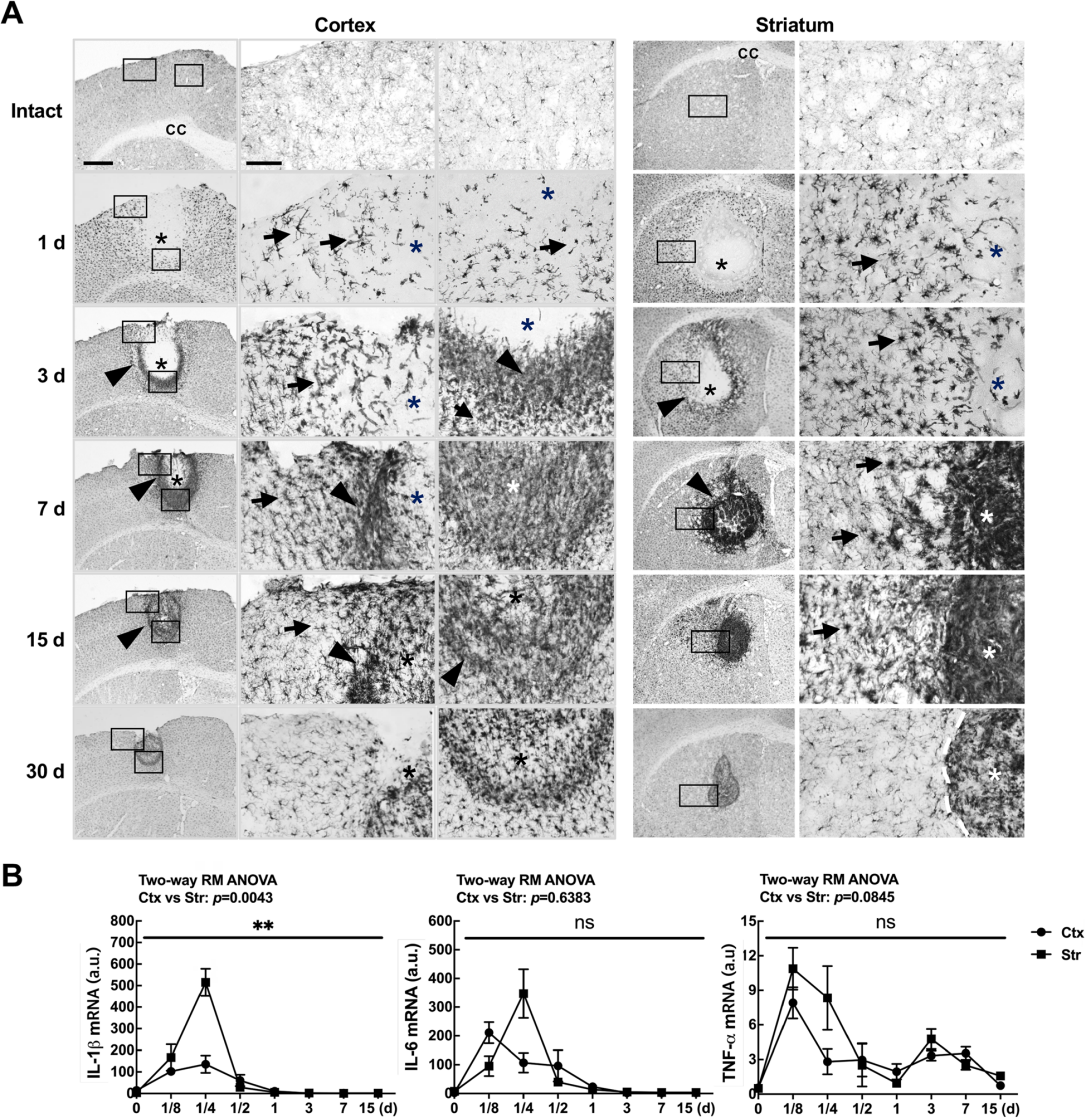
Inflammatory responses in the injured cortex and striatum. **a** Sections from the cortex and striatum were immunostained for Iba-1 at the indicated times post injection. Photographs of the most damaged sections are shown. Arrows, Iba-1-positive cells with a hypertrophic morphology; arrowheads, Iba-1–positive cells at the boundary of injury sites. **b** At the indicated times after ATP injection, IL-1β, IL-6, and TNF-α mRNA levels were analyzed using RT-qPCR. Values are means ± SEMs of 5 animals for both cortex and striatum (*, *p* < 0.05; ***, *p* < 0.001; ns, not significant; two-way ANOVA, IL-1β: F(7, 56) = 14.03, *p* < 0.0001, interaction; F(2.091, 16.73) = 39.77, p < 0.0001, time; F (1, 8)=15.52, *p* = 0.0043, region; F(8, 56) = 0.964, *p* = 0.4731, subjects; IL-6: F(7, 56) = 6.29, p < 0.0001, interaction; F(2.031, 16.10) = 17.41, p < 0.0001, time; F (1, 8)=0.2387, *p* = 0.6383, region; F(8, 56) = 1.515, *p* = 0.1728, subjects; TNF-a: F(7, 56) = 1.842, *p* = 0.0972, interaction; F(2.772, 22.17) = 12.03, p < 0.0001, time; F (1, 8)=3.876, *p* = 0.0845, region; F(8, 56) = 0.7877, *p* = 0.6154, subjects)

Next, we examined possible roles of inflammatory responses in differential astrogliosis because microglial activation and/or inflammation in the injured brain are important for astrogliosis [3, 5, 29]. In the intact brain, Iba-1–positive resident microglia were evenly distributed in both cortical and striatal regions (Fig. 5a intact). As we previously reported [21], microglia died in the injury core demonstrated by the absence of Iba-1 immunoreactivity at 1 and 3 d (*, Fig. 5a). From 1 to 30 d post-injury, Iba-1–positive cells became hypertrophic in the penumbra regions (arrows in Fig. 5a), and filled the damage core from the edge in both regions (arrowheads in Fig. 5a). Interestingly, Iba-1–positive cells were more densely packed in the striatum at 7 and 15 d (Fig. 5a). At 30 d, the density of these cells in the core was decreased in both regions, and their morphology in the penumbra region became less hypertrophic (Fig. 5a). Notably, the area surrounded by and/or filled with Iba-1–positive cells decreased at later time points as GFAP-negative areas decreased (Fig. 5a vs. Fig. 3a). We also examined mRNA levels of the inflammatory cytokines, IL-1β, IL-6, and TNF-α in the injured cortex and striatum. The mRNA levels of IL-1β and IL-6 peaked 3–6 h after ATP injection and then decreased to basal levels within 24 h (Fig. 5b), but that of TNF-α rather slowly decreased to 15 d in both regions (Fig. 5b). In general, the expression levels of these cytokines were higher in the injured striatum than in the injured cortex (Fig. 5b).

**Fig. 6:**
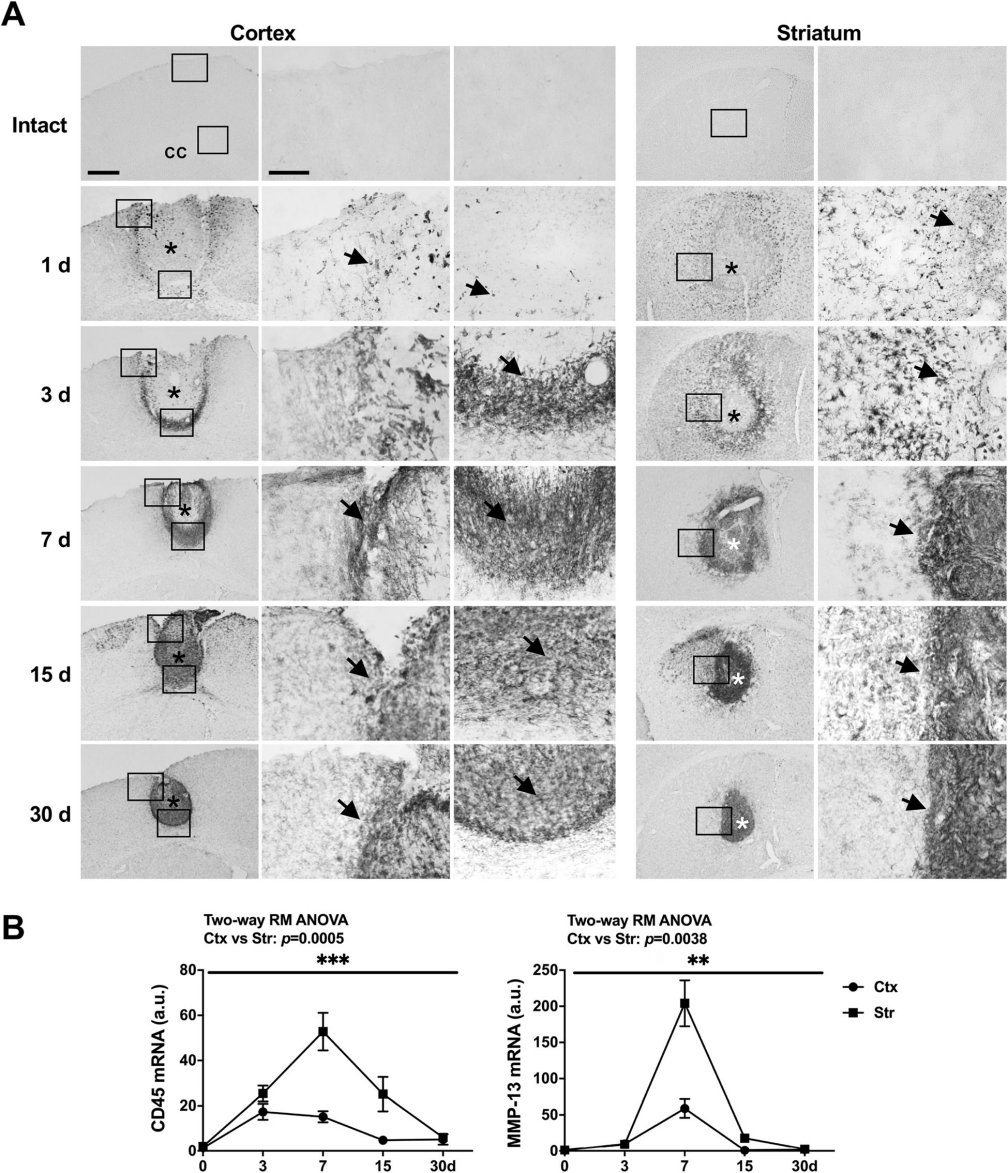
Time-dependent behavior of CD45-positive cells in the ATP-injured cortex and striatum. **a** Sections were obtained from the cortex and striatum at the indicated times post-ATP injection, and stained for CD45. CD45-positive cells were not detected in the intact brain, but appeared at the boundary of injury sites 1 d post-injury (arrows), and gradually filled the injury core at 3–30 d (arrows). Like Iba-1–positive cells, CD45-positive cells were more densely packed in areas surrounding the damage sites, and near the corpus callosum in the damaged cortex and striatum. **b** CD45 and MMP-13 mRNA levels were analyzed by RT-qPCR at the indicated times after ATP injection. Values are means ± SEMs of at least 3 animals (*, *p* < 0.05, **, *p* < 0.001; two-way ANOVA, CD45: F(4, 40) = 7.336, *p* = 0.0002, interaction; F(1.949, 19.49) = 19.87, p < 0.0001, time; F (1, 10)=26.01, *p* = 0.0005, region; F(10, 40) = 1.046, *p* = 0.4245, subjects; MMP-13: F (4, 32)=18.23, p < 0.0001, interaction; F(1.044, 8.349) = 57.95, p < 0.0001, time; F (1, 8)=16.19, *p* = 0.0038, region; F (8, 32)=1.489, *p* = 0.2002, subjects)

Next, we examined infiltration of monocytes using antibodies specific for CD45, which is more highly expressed in monocytes than in microglia although Iba-1 is similarly expressed in microglia and monocytes [17, 21, 22]. CD45-positive cells were not detectable in intact brain, appeared at the boundaries of injury sites from 1 d after the injury, and progressively filled the injury core at later time points in both regions (arrows in Fig. 6a). In QPCR analysis, CD45 mRNA levels were higher in the striatum than in the cortex (Fig. 6b). We also examined mRNA levels of MMP-13 since it has been reported that MMP-13 is produced by monocytes and degrades CSPGs, a component of scar [12, 13]. Similar to CD45, mRNA levels of MMP-13 were also higher in the striatum than in the cortex (Fig. 6b). Taken together, these results suggest that different patterns of astrogliosis in the cortex and striatum may be caused by factor(s) other than astrocyte proliferation and inflammatory cytokines. In addition, monocyte infiltration may negatively affect scar formation through production of proteases.

### Blood vessels and/or perivascular cells contribute to scar formation in the cortex near the meninges

**Fig. 7:**
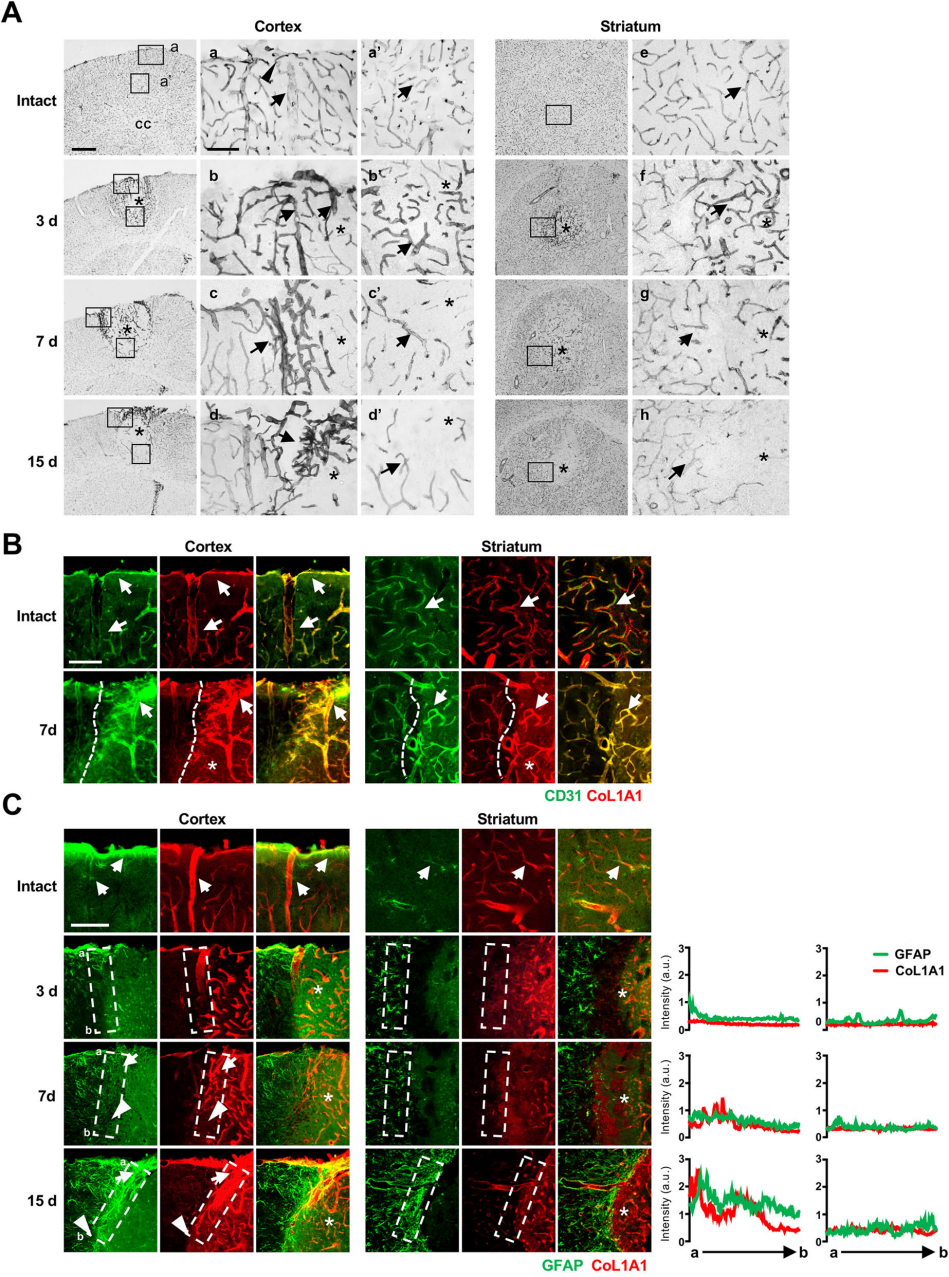
Region-specific blood vessel formation and locations of perivascular cells in the injured brain. **a** Sections obtained from the intact and ATP-injected cortex and striatum at the indicated times were immunostained for CD31 to label blood vessels. Arrowheads, vessels located along the meninges in the intact cortex; arrows, vessels in the parenchyma in intact and injured brains. **b** Colocalization of CD31 and Col1a1. Sections from intact and ATP-injected brains were double-labeled with antibodies for CD31 and Col1a1 (perivascular fibroblasts), and visualized using Alexa 488- and 594-conjugated secondary antibodies. CD31 and Col1a1 were colocalized in the intact and injured cortex and striatum (arrows). Dotted lines, edges of the damage areas. **c** Sections were double-labeled with antibodies for GFAP and CoL1A1. Arrows, astrocytes near blood vessels and/or in vessel-rich regions; arrowheads, astrocytes in vessel-rare regions. Relative intensities of GFAP and CoL1A1 in the regions of interest (white boxes) were plotted

We further examined the factors that contribute to scar formation in injured cortex. Blood vessels are likely candidates, given that several components of glial scars are produced by blood vessels and/or cells surrounding them [7, 11, 30, 31]. In addition, large blood vessels are located in the cortex, particularly near meninges [20]. On the basis of these observations, we examined the locations of blood vessels in the cortex and striatum using an antibody against CD31, a marker of endothelial cells [32]. In the intact cortex, blood vessels were located along the meninges (arrowheads in Fig. 7Aa), and large vessels were found in the cortex near the meninges (arrows in Fig. 7Aa) than in the cortex near the corpus callosum (arrows in Fig. 7Aa’) or in the striatum (arrows in Fig. 7Ae). At 3 d post-ATP injection, CD31 levels were increased in the damaged areas in both the cortex and striatum (arrows in Fig. 7Ab, b’, f). However, at 7 and 15 d post-ATP injection, region-specific changes in blood vessels became apparent: blood vessels were densely packed in the boundary of damaged areas in the cortex near the meninges (arrows in Fig. 7Ac, d), but not in the cortex near the corpus callosum (arrows in Fig. 7Ac’, d’) or in the striatum (arrows in Fig. 7Ag, h).

It has been reported that type 1 collagen produced by pericytes is important for scar formation [11]. Thus, we examined spatial and temporal correlations among the locations of blood vessels and astrocytes. Pericytes were colocalized with blood vessels in the cortex and striatum in both intact and injured brains, as revealed by double labeling with antibodies for CD31 and the pericyte marker, CoL1A1 (arrows in Fig. 7b). In the intact cortex and striatum, GFAP immunoreactivity was found in astrocytes near meninges, blood vessels, as well as in the parenchyma (Fig. 7c, intact). At 3–15 d after the injury, GFAP and CoL1A1 immunoreactivity were detectable in both the cortex and striatum (arrows in Fig. 7c). It is noticeable that in the cortex near the meninges, density of CoL1A1 immunostaining was higher 7 and 15 d post injury (arrows in Fig. 7c, d and 15 d) compared with that in the cortex near the corpus callosum (arrowheads in Fig. 7c, d and 15 d).

**Fig. 8:**
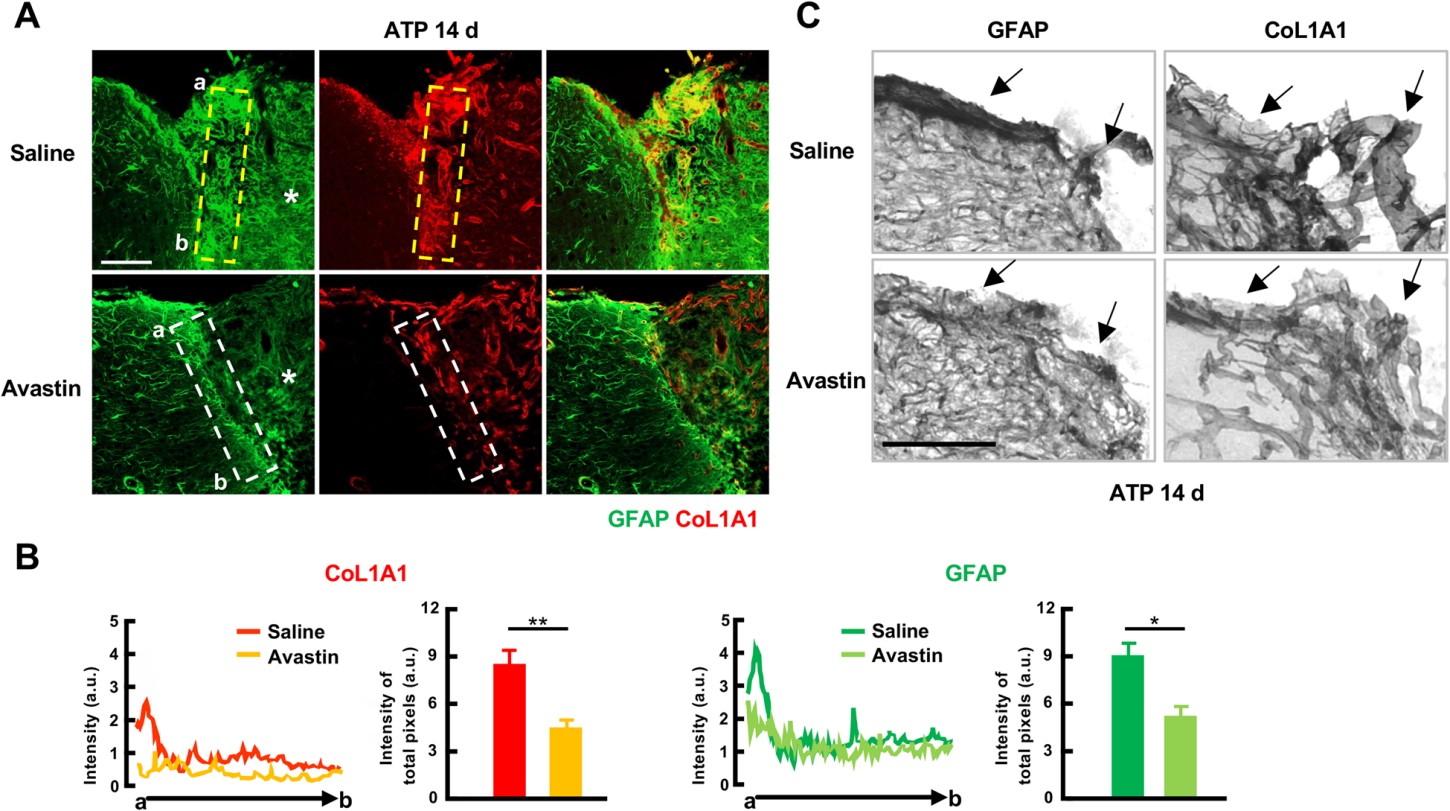
Avastin attenuates astrogliosis and blood vessel formation in the injured cortex. **a, c** Cortical sections were obtained from mice in which Avastin or PBS was infused into the ventricle 14 d post-ATP injection. GFAP and CoL1A1 were visualized with Alexa-488 and Alexa-594 conjugated secondary antibodies (**a**) or biotinylated secondary antibodies and DAB-based color reaction (**c**). **b** Relative fluorescence intensity of GFAP and CoL1A1 were measured using ZEN software and plotted. Values are means ± SEMs for animals treated with saline (*n* = 4) or Avastin (*n* = 3) (*, *p* < 0.05; Student’s t-test, GFAP: F (3, 4)=7.983, *p* = 0.0017; CoL1A1: F (3, 5)=1.638, *p* = 0.0185)

We further examined the effect of blood vessel formation on dense astrogliosis in the cortex. For this, vessel formation was blocked using the anti-VEGF antibody, Avastin [26], delivered to the ventricle immediately after induction of injury using minipump-delivered ATP. In Avastin-treated animals 14 d post injury, the density of GFAP-positive astrocytes in the cortex near meninges was attenuated together with a decrease in blood vessel formation, as confirmed by a reduction in CoL1A1 immunoreactivity (Fig. 8a, b, c). Taken together, these results suggest that vessel formation is critical for the development of dense astrogliosis and/or glia scar, leading to different patterns of astrogliosis in the cortex and the striatum.

## Discussion

Emerging evidence indicates that astrocytes are heterogeneous in different brain regions and even within the same brain region [14, 16, 19, 33, 34]. In the current study, we demonstrated that astrogliosis develops differently in different brain regions. In particular, scars and/or scar-like dense astrogliosis formed in the cortex near the meninges, whereas relatively loose astrogliosis formed in the striatum and cortex near the corpus callosum. Notably, blood vessel formation was found to be a critical factor for scar formation and region-specific astrogliosis.

Astrogliosis is a common phenomenon in the injured brain. However, the patterns of astrogliosis were different in the cortex and the striatum (Fig. 3). Even in the cortex, dense astrogliosis formed near the meninges, but not near the corpus callosum (Fig. 3a). Accordingly, damage in the cortex in the absence of meninges damage produced astrogliosis similar to that in the injured cortex near the corpus callosum and striatum (Fig. 3b).

It has been reported that the severity of damage determines the reversibility of astrogliosis, with irreversible scars forming in the severely injured brain [3]. However, the severity of the injury may not be enough to cause scar-like astrogliosis in the cortex, since ATP induced less damage in the cortex than in the striatum (Fig. 2a), although at this point, we do not have a clear explanation for why the same amount of ATP induced less damage in the cortex than in the striatum. In addition, GFAP-positive and -negative cell proliferation, which is known to contribute to astrogliosis [6, 27, 28], may not be the factor that induces differences in astrogliosis in the cortex and the striatum. In the current study, Ki67-immunoreactivity was detectable in both GFAP-positive and GFAP-negative cells in the core and penumbra regions of cortex and striatum (Fig. 4a, b). However, there was little difference in the number of Ki67-positive cells in cortex scar-forming regions and other brain regions (Fig. 4a, b). Although it has been reported that inflammatory mediators such as IL-1β play a role in astrogliosis by enhancing proliferation of astrocytes and expression of astrogliosis components [3, 5, 29], levels of all inflammatory mediators tested (IL-1β, TNF-α, and IL-6) were somewhat lower in the cortex than in the striatum (Fig. 5b). Therefore, inflammatory cytokines may not affect regional differences in astrogliosis. Interestingly, monocytes inhibit accumulation of glial scar components, including CSPGs, through production of MMP-13 [12, 13], indicating that inflammation also exerts a negative influence on astrogliosis. In this study, we found that Iba-1 and/or CD45 immunoreactivity in the cortex near the meninges was lower than that in the cortex near the corpus callosum or the striatum (Figs. 5a and 6a). In addition, MMP-13 and CD45 levels were higher in the striatum than in the cortex (Fig. 6b).

Blood vessels and/or perivascular cells appeared to be important regulators of region-specific astrogliosis (Fig. 7). It has recently has been reported that type 1 collagen plays a critical role in converting reactive astrocytes into scar-forming astrocytes [11]. In addition, it has been reported that pericytes, a major source of collagen [30, 35], play active roles in astrogliosis and/or scar formation [36, 37]. Consistent with this, in this study, we found that scar-like astrogliosis was formed in the cortex near the meninges where CoL1A1-positive vessel formation was prominent (Fig. 7), and astrocytes converged on vessels (Fig. 7). The importance of vessels in scar formation was additionally confirmed by administration of Avastin, which inhibited formation of scar-like astrogliosis as well as vessel formation (Fig. 8). However, we cannot exclude the possibility that Avastin directly acts on astrocytes to attenuate astrogliosis, since VEGF regulates astrocyte proliferation [38]. Collectively, these results suggest that blood vessel formation plays a critical role in the development of scar-like astrogliosis in the cortex near meninges, although the severity of damage, proliferation of astrocytes, and inflammatory cytokines are important for astrogliosis.

Our results raise the following questions: Why are blood vessels more highly formed in the injured cortex near the meninges? And what is the physiological importance of different patterns of astrogliosis in different brain regions? In the intact brain, blood vessels are densely located between the pia mater and the subarachnoid space and supply blood to the entire cortex [39]. Therefore, regeneration of blood vessels near the meninges may be critical for supplying the remaining brain regions with blood. In addition, dense astrogliosis and/or glia scar may be required for formation of new meninges in the injured brain.

## Data Availability

Please contact the author for data and materials requests.

## Abbreviations

ATP: Adenosine triphosphate
MR: Magnetic resonance
VEGF: Vascular endothelial growth factor
ALDH1L1: Aldehyde Dehydrogenase 1 Family Member L1
GFAP: Glial fibrillary acidic protein
NG2: Neuron glia antigen-2
Col1a1: Collagen 1a1
CSPG: Chondroitin sulfate proteoglycan
MMP-13: Matrix metalloproteinase 13
Ki-67: Cell proliferation antigen Ki-67
GAPDH: Glyceraldehyde 3-phosphate dehydrogenase
CD45: Cluster of differentiation 45
CD31: Cluster of differentiation 31
Iba-1: Ionized calcium binding adaptor molecule 1
MAP2: Microtubule associated protein 2
TH: Tyrosine hydroxylase
DAPI: 4′,6-diamidino-2-phenylindole
HRP: Horseradish peroxidase
IL-1β: Interleukin 1 beta
IL-6: Interleukin 6
TNF-α: Tumor necrosis factor-alpha
PBS: Phosphate-buffered saline
Ctx: Cortex
Str: Striatum
Hippo: Hippocampus
Cc: Corpus callosum
Mid: Midbrain
Ob: Olfactory bulb
Cb: Cerebellum
AP: Anterior- Posterior
ML: Medial-Lateral
DV: Dorsal-Ventral
DAB: 3,3′-diaminobenzidine
qPCR: Quantitative real-time polymerase chain reaction
